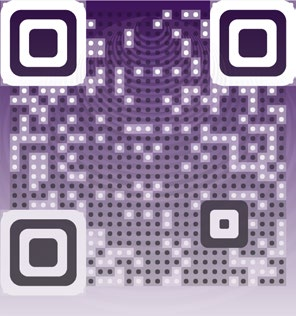# Continuing Professional Development—Radiation Therapy

**DOI:** 10.1002/jmrs.883

**Published:** 2025-05-02

Maximise your continuing professional development (CPD) by reading the following selected article and answering the five questions. Please remember to self‐claim your CPD and retain your supporting evidence. Answers will be available via the QR code and published in JMRS—Volume 72, Issue 4, December 2025.

## An Evaluation of Treatment Time and Intrafraction Motion in Stereotactic Body Radiation Therapy

Leila Rough, Julie Burbery, Catriona Hargrave, Elizabeth Brown, *Journal of Medical Radiation Sciences* (2025), https://doi.org/10.1002/jmrs.861.

1. What is Intrafraction Imaging (IFI) as described in this article?
  - IFI is a form of mid‐treatment imaging acquired in between treatment arcs to confirm and correct the patient's position
  - IFI refers to the use of kV cone‐beam computed tomography (CBCT) images taken simultaneously with arc delivery to confirm and correct the patient's position
  - IFI refers to MV cone‐beam computed tomography (CBCT) images taken during arc delivery to confirm the patient is in the correct treatment position
  - IFI is a method to reduce the chance of a patient moving during radiation treatment
2. According to this article, what stages of the treatment process does ‘treatment time’ refer to?
  - From when the patient enters the room until they leave the room
  - The duration the beam is on
  - From the moment the first image is taken to the end of the final IFI image acquisition
  - The time it takes to set up the patient in their treatment position
3. In which treatment site were the longest treatment times and the largest intrafraction motions observed?
  - Lung
  - Liver
  - Spine
  - Both liver and lung sites exceeded the clinical threshold of 0.3 cm and showed equal treatment durations
4. Which of the following factors was not highlighted in the study as contributing to intrafraction motion in patients with SBRT?
  - Patients may have comorbidities that affect their ability to remain still
  - Patients required to perform breath hold techniques may show deviations in breath hold reproducibility
  - The specific treatment site receiving radiation may exhibit different levels of intrafraction motion
  - Whether the patient had prior treatment before their current treatment course
5. At what stage of imaging was intrafraction motion most identified and corrected?
  - Upon review of the confirmation image, prior to treatment
  - During the IFI from the first treatment arc
  - Upon review of the localisation image, prior to treatment
  - During the IFI from the last treatment arc

## Answers

Scan this QR code to find the answers.